# Identification and characterization of microRNAs related to salt stress in broccoli, using high-throughput sequencing and bioinformatics analysis

**DOI:** 10.1186/s12870-014-0226-2

**Published:** 2014-09-03

**Authors:** Yunhong Tian, Yunming Tian, Xiaojun Luo, Tao Zhou, Zuoping Huang, Ying Liu, Yihan Qiu, Bing Hou, Dan Sun, Hongyu Deng, Shen Qian, Kaitai Yao

**Affiliations:** Cancer Research Institute, Southern Medical University, Guangzhou, Guangdong Province People’s Republic of China; Department of Oncology, Armed Police Hospital of Guangdong Province, Guangzhou, Guangdong Province People’s Republic of China; State Key Laboratory of Oncology of Southern China, Guangzhou, Guangdong Province People’s Republic of China; Department of Radiation Oncology, Cancer Center of Sun Yat-Sen University, Guangzhou, Guangdong Province People’s Republic of China

**Keywords:** Broccoli, Salt stress, High-throughput sequencing, microRNA

## Abstract

**Background:**

MicroRNAs (miRNAs) are a new class of endogenous regulators of a broad range of physiological processes, which act by regulating gene expression post-transcriptionally. The brassica vegetable, broccoli (*Brassica oleracea var. italica*), is very popular with a wide range of consumers, but environmental stresses such as salinity are a problem worldwide in restricting its growth and yield. Little is known about the role of miRNAs in the response of broccoli to salt stress. In this study, broccoli subjected to salt stress and broccoli grown under control conditions were analyzed by high-throughput sequencing. Differential miRNA expression was confirmed by real-time reverse transcription polymerase chain reaction (RT-PCR). The prediction of miRNA targets was undertaken using the Kyoto Encyclopedia of Genes and Genomes (KEGG) Orthology (KO) database and Gene Ontology (GO)-enrichment analyses.

**Results:**

Two libraries of small (or short) RNAs (sRNAs) were constructed and sequenced by high-throughput Solexa sequencing. A total of 24,511,963 and 21,034,728 clean reads, representing 9,861,236 (40.23%) and 8,574,665 (40.76%) unique reads, were obtained for control and salt-stressed broccoli, respectively. Furthermore, 42 putative known and 39 putative candidate miRNAs that were differentially expressed between control and salt-stressed broccoli were revealed by their read counts and confirmed by the use of stem-loop real-time RT-PCR. Amongst these, the putative conserved miRNAs, miR393 and miR855, and two putative candidate miRNAs, miR3 and miR34, were the most strongly down-regulated when broccoli was salt-stressed, whereas the putative conserved miRNA, miR396a, and the putative candidate miRNA, miR37, were the most up-regulated. Finally, analysis of the predicted gene targets of miRNAs using the GO and KO databases indicated that a range of metabolic and other cellular functions known to be associated with salt stress were up-regulated in broccoli treated with salt.

**Conclusion:**

A comprehensive study of broccoli miRNA in relation to salt stress has been performed. We report significant data on the miRNA profile of broccoli that will underpin further studies on stress responses in broccoli and related species. The differential regulation of miRNAs between control and salt-stressed broccoli indicates that miRNAs play an integral role in the regulation of responses to salt stress.

**Electronic supplementary material:**

The online version of this article (doi:10.1186/s12870-014-0226-2) contains supplementary material, which is available to authorized users.

## Background

MicroRNAs (miRNAs) are a class of non-coding small RNAs (sRNAs), approximately 20–24 nucleotides in length, that post-transcriptionally regulate gene expression. In plants, highly conserved and species-specific miRNAs control a vast array of biological processes, such as leaf polarity, flower development and stress responses [1,2]. The miRNAs are excised from stem-loop structures within larger primary miRNA transcripts by Dicer-Like1 (DCL1), which in each case trims the hairpin structure (pre-miRNA). In plants, the mature miRNA strands have high complementarity (fewer than four mismatches) to their target mRNAs and regulate gene expression via mRNA cleavage [3-5].

Analysis indicates that many plant miRNAs and their targets are evolutionarily conserved from species to species within the plant kingdom. Thus, a miRNA in one species may exist as orthologs or homologs in other species [6,7]. Plant miRNAs have usually been identified either by prediction through bioinformatics or by experimental methods [8]. However, the bioinformatics-based approaches can only identify miRNAs that are conserved amongst organisms, and DNA or RNA sequence information is required in order to run the programs. Thus, sequencing is the most effective method for plant miRNA discovery [9]. To date, 7384 sequences of mature miRNAs have been identified in plants, including 337 from *A. thaliana*, 384 from *A. lyrata*, 713 from *O. sativa* and 43 from *B. rapa* (miRBase release 20.0, June, 2013, http://mirbase.org/).

The genus *Brassica* is cultivated in most parts of the world. It includes various crops of agronomic importance, such as broccoli, cauliflower and *B. rapa*. Broccoli (*Brassica oleracea var. italica*) is very popular with a wide range of consumers because of its flavor, and also on account of its anti-cancer activities, notably in relation to prostate and colorectal cancers [10]. Because broccoli, *A. thaliana* and *B. rapa* all belong to the same family, the Cruciferae, the level of synteny between these species provides a basis for studying the miRNAs of broccoli [11,12].

Environmental stresses such as salinity are a worldwide problem in agriculture, diminishing plant growth and yield. Broccoli is moderately tolerant to salinity, and it displays better tolerance than some other common vegetables, such as maize and carrot [13,14]. Although several stress-related miRNAs have been identified based on the sequencing of a library of sRNAs isolated from *A. thaliana* seedlings, *O. sativa*, *saccharum officinarum L.* and from *populous euphratica* exposed to various stresses [15-19], there have been few studies that have focused upon miRNAs in broccoli, and in particular upon the identification of stress-related miRNAs in broccoli.

Thus, in the present study, we have identified miRNAs and their targets related to salt-stress in broccoli, using high-throughput sequencing methods. The differential expression of miRNAs observed between broccoli grown under standard conditions and broccoli subjected to salt stress provides new insights that will inform the genetic improvement of stress tolerance in plants.

## Results

### Sequence analysis of sRNAs

**Figure 1:**
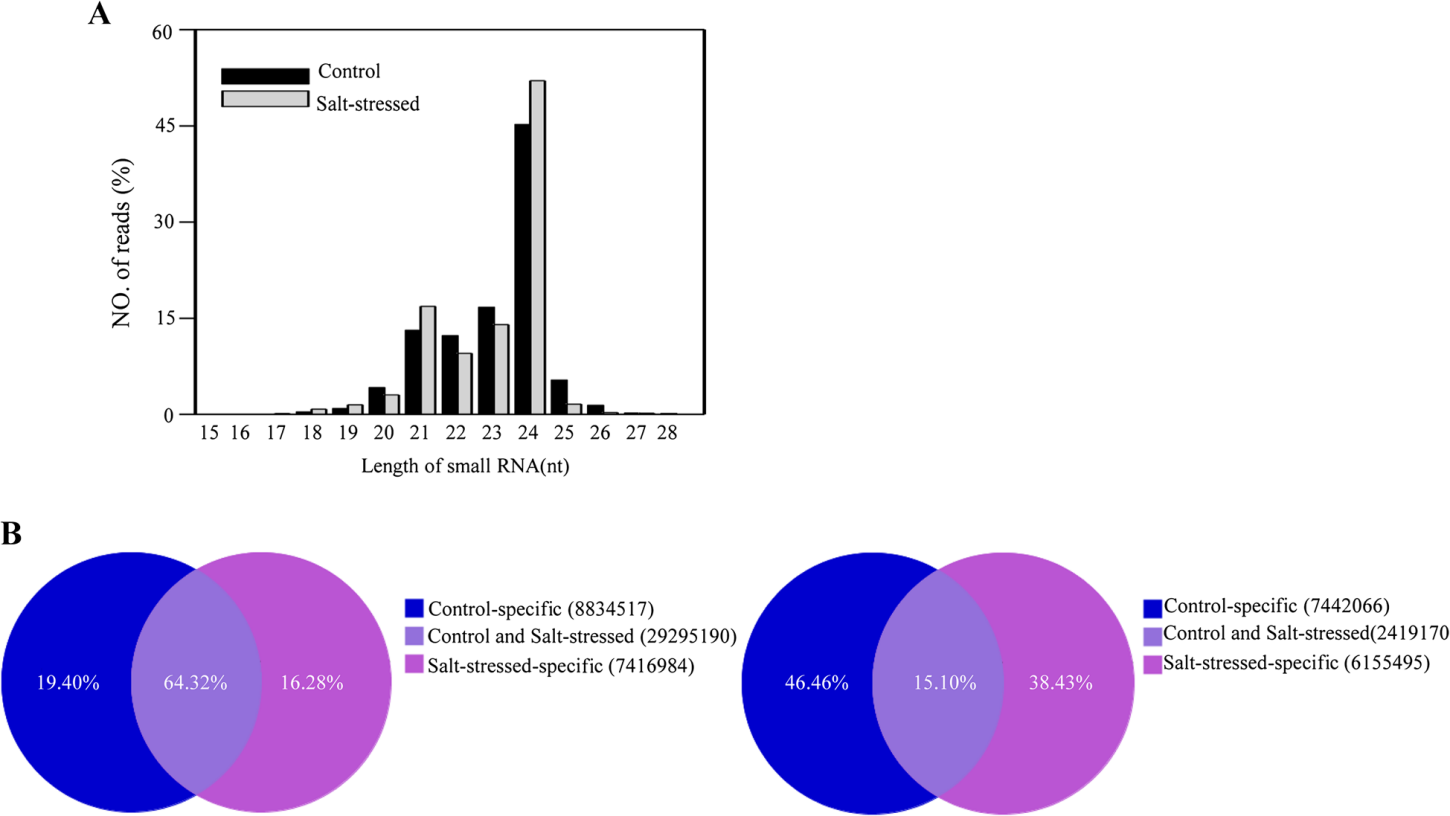
**Sequence analysis of sRNAs. (A)** Length distribution of sRNAs. The length distribution of high-quality sequences obtained from the two broccoli libraries. The distributions of the total reads are shown as percentages. **(B)** Summary of common and specific sequences between control and salt-stressed broccoli libraries. Total sRNAs and unique sRNAs are shown in the left panel and the right panel, respectively.

In order to identify the miRNAs responding to salt stress, we constructed and sequenced sRNA libraries ranging in size from 18 to 30 nt, from both control and salt-stressed broccoli. A total of 24,655,210 and 21,196,508 reads were obtained from control and salt-stressed broccoli, respectively. After removing the tags with: any N bases, more than 4 bases whose quality score was lower than 10 and more than 6 bases whose quality score was lower than 13, and those that were too small (with length shorter than 18 nt), as well as the adapter sequences, 24,511,963 (99.74%) and 21,034,728 (99.56%) clean reads were obtained (Additional file 1: Table S1), representing 9,861,236 (40.23%) and 8,574,665 (40.76%) unique, although sometimes partially overlapping, clean reads from control and salt-stressed broccoli, respectively. The size distributions of the reads in the two datasets were quite similar, and they were not evenly distributed. The majority of these unique sequence reads were 24 nt in size, followed by 23 nt, 21 nt and 22 nt, both in control and salt-stressed broccoli, which is similar to the size distribution of sRNAs in plants (Figure 1A). The overall distribution pattern of sRNAs (21 nt sRNAs = 16.81%, and 24 nt sRNAs = 52.01%) in salt-stressed broccoli was similar to that in control broccoli (21 nt sRNAs = 15.23%, and 24 nt sRNAs = 48.81%). Moreover, common and specific tags between the control and salt-stressed broccoli were analyzed. The results showed that only 2,419,170 (15.10%) of the unique sequences and 29,295,190 (64.32%) of the total sequences were shared between the two samples (Figure 1B), suggesting that the sequence results were reliably representative of the endogenous sRNAs in broccoli.

Because the whole genome of sequence for broccoli was not available, all clean reads were aligned against the *B. rapa* genome, using short oligonucleotide alignment program-2 (SOAP2, http://soap.genomics.org.cn) [20]. However, only a very small percentage of the unique sRNA sequences could be matched against the *B. rapa* genome; only 1,491,240 (15.12%) of the reads in the control broccoli and 1,276,113 (14.88%) of the reads in the salt-stressed broccoli could be mapped in this way, representing 7,163,496 (29.22%) and 6,168,917 (29.33%) of the total reads in control and salt-stressed broccoli, respectively (Additional file 2: Table S2). All clean reads were annotated into different categories, including plant miRNAs (miRbase, http://www.mirbase.org/), exons and introns (*B. rapa* genome, http://www.ncbi.nlm.nih.Gov/genbank), and non-coding RNAs (Rfam, http://www.sanger.ac.uk). In those cases in which sRNAs were mapped to more than one category, the following priority rule was adopted: rRNA > known miRNA > exon > intron. The results indicated that the majority of sRNAs – 91.90% of the unique reads in the control group and 92.23% in the salt-stressed group – remained unannotated. For the control group, when unique sRNAs were matched, a small proportion of reads were derived from repeated sequences (3.98%), and a smaller proportion from rRNAs (1.39%). However, for the total sRNA pools, rRNAs (7.79%) were the most abundant sequences, followed by repeated sequences (5.72%) (Table 1; Additional file 3: Figure S1). All the unannotated sequences were then used for further analysis.

**Table 1:** **Distribution of genome-mapped sequence reads in the sRNA libraries for control and salt-stressed broccoli**

**Locus class**	**Control**				**Salt-stressed**			
	**Unique sRNA**	**Percent (%)**	**Total sRNA**	**Percent (%)**	**Unique sRNA**	**Percent (%)**	**Total sRNA**	**Percent (%)**
Total	9861236	100%	24511963	100%	8574665	100%	21034728	100%
Exon antisense	33857	0.34%	91879	0.37%	39594	0.46%	83220	0.40%
Exon sense	56641	0.57%	168950	0.69%	61307	0.71%	138900	0.66%
Intron antisense	60209	0.61%	215558	0.88%	53351	0.62%	168011	0.80%
Intron sense	88950	0.90%	406032	1.66%	78075	0.91%	331915	1.58%
miRNA	6595	0.07%	810592	3.31%	6385	0.07%	1053521	5.01%
rRNA	137427	1.39%	1909705	7.79%	114451	1.33%	1807910	8.59%
repeat	392888	3.98%	1402050	5.72%	295752	3.45%	904109	4.30%
snRNA	4766	0.05%	13349	0.05%	5028	0.06%	13861	0.07%
snoRNA	3159	0.03%	8316	0.03%	2250	0.03%	4588	0.02%
tRNA	14221	0.14%	300106	1.22%	10021	0.12%	192288	0.91%

### Identification and expression patterns of salt-stress-induced conserved miRNAs in broccoli

**Figure 2:**
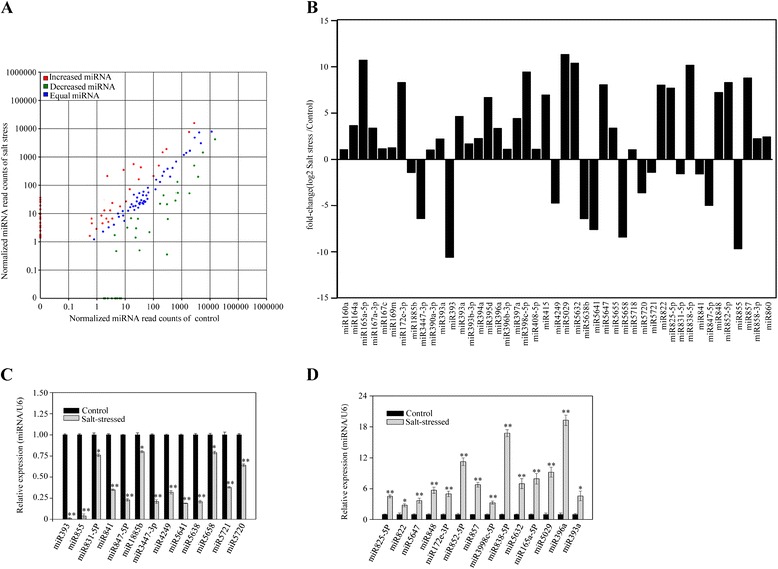
**Differential expression of putative conserved miRNAs between control and salt-stressed broccoli. (A)** Scatter diagram of the differential read counts of known miRNAs. Each point in the figure represents a miRNA. Red points represent miRNAs showing a > 2-fold change of expression; blue points represent miRNAs showing 1/2 < fold change ≤ 2; green points represent miRNAs showing a fold change ≤ 1/2. **(B)** The 45 miRNAs showing the greatest changes in expression, with fold changes < 1/2 or > 2. Decreased **(C)** and increased **(D)** putative conserved miRNAs confirmed by stem-loop real-time RT-PCR, as described in Methods. Significant difference between salt-stressed broccoli and control broccoli is indicated by *P < 0.05 and **P < 0.01.

Conserved miRNA families are found in many plant species and play an important role in a diversity of plant processes. In spite of this, sequence information for broccoli miRNAs is absent from both the miRBase database and from the plant miRNA database. Furthermore, although miRNA families are conserved between closely related species [6,21], there are only 43 known *B. rapa* miRNAs in miRBase. Therefore, the sRNA sequences were aligned to the miRNA precursor/mature miRNA sequences of the Viridiplantae in miRBase. Ninety-seven putative known miRNAs were identified in salt-stressed broccoli, including 72 miRNAs that were also found in the control group (Additional file 4: Table S3). In particular, the putative conserved miRNAs, miR156a, miR166a and miR168a, which have been found in about 40 plant species (miRBase release 20.0), were amongst the miRNAs that were found in both groups. A comparison of the miRNAs in the two libraries indicated that about 40 putative miRNAs – including, for example, miR393, miR841 and miR5658 – were less abundant in salt-stressed broccoli than in control broccoli. Conversely, 45 miRNAs – including, for example, miR157d, miR393b-3p and miR169m – were more abundant in salt-stressed broccoli than in control broccoli (Figure 2A). Although the levels of some miRNAs belonging to the same miRNA gene family, such as miR157, miR158 and miR160, were found to be increased and decreased in parallel, others were not; the levels of miR393 and miR393a, for example, increased and decreased differently (Additional file 4: Table S3). However, when the fold-change (log2) criterion between salt-stressed broccoli and control broccoli was set to < −1.0 or > 1.0, and the false discovery rate (FDR) was set to < 0.01, the levels of 45 miRNAs were found to be significantly different between salt-stressed and control plants. As shown in Figure 2B, the levels of miR393, miR855, miR841, miR168a and miR5641 were all decreased; levels of miR160, miR396a, miR397a and miR164a, on the other hand, were increased under salt stress. The miRNA that showed the greatest decrease under salt stress was miR393 and the miRNAs that showed the greatest increase were miR165a-5P and miR5029.

Real-time quantification of miRNAs by stem-loop real-time reverse transcription polymerase chain reaction (RT-PCR) is specific for mature miRNAs and discriminates amongst related miRNAs that differ by as little as one nucleotide. Furthermore, it is not affected by genomic DNA contamination [22,23]. Therefore, this technique was used to validate the sequencing results. To confirm the identity of the individual PCR products, they were firstly confirmed by electrophoretic sequencing and by denaturing gel electrophoresis. For those miRNAs that were less abundant in salt-stressed broccoli than in control broccoli, the results obtained by stem-loop RT-PCR agreed with the results obtained by sequencing. The greatest degree of down-regulation in response to salt stress was shown by miR393 and miR855 (Figure 2C). Amongst the 32 miRNAs that were more abundant in salt-stressed broccoli than in control broccoli, 29 also showed up-regulated expression when analyzed by stem-loop RT-PCR; the three exceptions were miR5655, miR858-3P and miR415, which were not detected by this technique, either in control or in salt-stressed broccoli. Of these 29, miR396a showed the greatest degree of up-regulation, followed by miR838-5P, miR852-5P, miR848 and miR393a (Figure 2D). These findings contrasted with the results obtained by sequencing, which indicated that the two most abundant up-regulated miRNAs were miR165a-5P and miR5029. In summary, 42 putative conserved miRNAs that were differentially expressed between control and salt-stressed broccoli were revealed by their read counts and confirmed by the use of stem-loop real-time RT-PCR.

### Identification and expression patterns of salt-stress-induced, non-conserved miRNAs in broccoli

**Figure 3:**
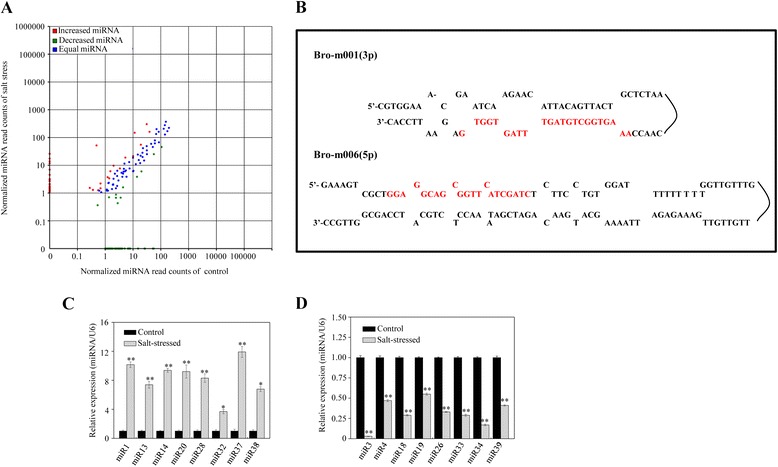
**Differential expression of putative novel miRNAs between control and salt-stressed broccoli. (A)** Scatter diagram of differential read counts of putative miRNAs. **(B)** Prediction of secondary structure of representative putative miRNAs from broccoli with or without salt treatment. Increased **(C)** and decreased **(D)** putative novel miRNAs confirmed by stem-loop real-time RT-PCR. Significant difference between salt-stressed broccoli and control broccoli is indicated by *P < 0.05 and **P < 0.01.

In order to identify putative novel miRNAs in the broccoli dataset, secondary structures and minimum free-energies were calculated. In total, 326 unique mature miRNAs were identified initially in our study, and these candidate miRNAs originated from predicted RNA hairpins. With an average of about 46 read counts, these putative novel miRNAs showed fewer read counts than the putative conserved miRNAs. A comparison of the putative novel miRNAs in the two libraries indicated that the levels of about 59 of theses miRNAs were decreased in response to salt stress. Conversely, the levels of about 50 miRNAs were increased (Figure 3A). Real time RT-PCR was next performed for those miRNAs with an average of more than 46 read counts either in the salt stressed broccoli or control broccoli, and whose levels were found to be significantly different between salt-stressed and control plants, when the fold-change (log2) criterion between salt-stressed broccoli and control broccoli was set to < −1.0 or > 1.0, and the FDR was set to < 0.01. The results from the 52 miRNAs analyzed by real time RT-PCR indicated that only 43 of these candidate miRNAs were expressed in either the control or the salt-stressed group (Table 2). A higher proportion of the sequences were identified from the 5’-ends of the hairpins than from the 3’-ends. Secondary hairpin structures for representative miRNAs are shown in Figure 3B, and the secondary hairpin structures for all the candidate miRNAs are listed in Additional file 5: Figure S2.

**Table 2:** **Putative novel miRNAs in the two libraries**

**Novel ID**	**Location**	**Strand (+/−)**	**Energy (kcal/m**	**Sequence of 5P**	**Sequence of 3P**
Bro-m001	A01:146563:146640	+	−20	-	AAAGTGGCTGTAGTTTAGTGG TG
Bro-m002	A01:13726926:13727021	+	−40.1	TACACTAGTTGTGGACATGGAC	-
Bro-m003	A02:16145164:16145426	+	−54.6	TCAAAGAATTAGTAGATCGGT	-
Bro-m004	A02:16493715:16493784	+	−24.9	-	TCGGACGTTTGTGACGGATTT CC
Bro-m005	A02:21365800:21365890	+	−22.9	-	GTGGACACTCTATCTGAGGCT TA
Bro-m006	A02:2629827:2629950	-	−45.3	GGAGGCAGCGGTTCATCGATC	-
Bro-m007	A02:22803188:22803422	-	−39	-	AGGGATATAGTGTATGGAGTG CA
Bro-m008	A02:25162550:25162723	-	−32.04	-	AAGTGGAGTAGAGTATAATGC AG
Bro-m009	A02:25162550:25162724	+	−47	AAAGATCTGAACCGAACACGAA	-
Bro-m010	A02:25162550:25162725	+	−22.1	TAATCATGTTTAGACTTAGATC	-
Bro-m011	A02:25162550:25162726	-	−21.6	ACGGTACGGTTTTAACAGTTAT	-
Bro-m012	A02:25162550:25162727	-	−18.5	AGAGACGATTCTTAACTTTTCTT	-
Bro-m013	A02:25162550:25162728	-	−51.7	GATCGAGATCCTTTGGAGTTAGTCC	GCTTGCTAGATGGATCTTGGA CA
Bro-m014	A02:25162550:25162729	-	−21.9	-	TGCGGGGTACAAAGACGTTTG
Bro-m015	A02:25162550:25162730	+	−24.7	TCTGTCAAGTGGAATGTGGCTT	-
Bro-m016	A02:25162550:25162731	+	−47.3	GTTCGGACGCGGCGTCAGACAC	-
Bro-m017	A02:25162550:25162732	-	−18.1	TTATTTACTTTTGTTAGACTCCA	-
Bro-m018	A02:25162550:25162733	-	−32.4	TTGACCAGACGACTTATATTTT	-
Bro-m019	A02:25162550:25162734	-	−51.7	CGGTCTGTGATGGTACTAGTA	-
Bro-m020	A02:25162550:25162735	-	−34.7	TTTTGGAGTAATGAGTTGGTTTA	-
Bro-m021	A02:25162550:25162736	+	−104.8	-	TGGATTGGAACCACATAGGCC
Bro-m022	A02:25162550:25162737	+	−36.6	TGGACGACTTAGTAGATGACTT	-
Bro-m023	A02:25162550:25162738	+	−38	AATCGATATGGACTAATATGGT	-
Bro-m024	A02:25162550:25162739	-	−61.7	TTTGCGGTTGCGGACGGTTA	-
Bro-m025	A02:25162550:25162740	-	−56.1	TAGCCAAGGATGACTTGCCTG	-
Bro-m026	A02:25162550:25162741	-	−75.9	TCTGACTGCTTAGATGATTGCTT	-
Bro-m027	A02:25162550:25162742	-	−33.9	-	AGAGACGTCTCTTAGCTTTTT TA
Bro-m028	A02:25162550:25162743	-	−62.3	-	AGAAGAAACGTAGAAAACAC TT
Bro-m029	A02:25162550:25162744	+	−50.5	CGACAGAAGAGAGTGAGCAC	-
Bro-m030	A02:25162550:25162745	+	−84.1	ATATACTCTCAAGCATATCAA	-
Bro-m031	A02:25162550:25162746	-	−71.8	-	TTCCCACAAGAACGAAAACTC
Bro-m032	A02:25162550:25162747	+	−28.4	AATGGACAAATGGATATGGGCT G	-
Bro-m033	A02:25162550:25162748	+	−46.3	TGACAGAAAGAGAGGTGAGCA CG	-
Bro-m034	A02:25162550:25162749	+	−18.5	-	TTATTTTTTGTAGCTATTTTTT
Bro-m035	A02:25162550:25162750	+	−54.2	TAGCCAAGGATGACTTGCCTG	-
Bro-m036	A02:25162550:25162753	-	−78.8	-	TAAGGACCCAAGAGCAAGGA C
Bro-m037	A02:25162550:25162754	+	−28.3	CAAGAGATCTGCAGCATGGCGC A	-
Bro-m038	A02:25162550:25162755	+	−71.1	TCTTGTTTGATTGGTGCATGTCA	-
Bro-m039	A02:25162550:25162756	-	−105.6	CAAGCGCTCTTCTCACAGCTT	-
Bro-m040	A02:25162550:25162757	-	−46	-	ACGAACCATAGTCCGTCGGTA
Bro-m041	A02:25162550:25162758	+	−59.2	-	TTATTTTTTGTAGCTATTTTTT A
Bro-m042	A02:25162550:25162760	-	−22.17	AAAGGAAAGTTAGAAGACTTCT	-
Bro-m043	A02:25162550:25162761	-	−50.2	-	TAAGAAACTTGCAATGGAGGA CA

Some of the putative novel miRNAs showed particular expression profiles. Thus, eight were expressed only in libraries generated either from control or from salt-stressed broccoli: three (miR5, miR8, miR24 and miR31) were detected only in control broccoli, whilst five (miR6, miR10, miR12, miR30 and miR39) were found only in salt-stressed broccoli. Four other putative novel miRNAs (miR2, miR9, miR16 and miR29) exhibited similar expression levels in both libraries. Although most of the putative novel miRNAs were observed in both libraries, their expression could be significantly different; for example, miR32 showed four-fold higher expression in salt-stressed broccoli than in control plants. Amongst those that were significantly up-regulated, miR37 was up-regulated to the greatest extent, followed by miR1, miR14 and miR20 (Figure 3C). In contrast, miR3 and miR34 were the putative novel miRNAs that showed the largest down-regulation in response to salt stress (Figure 3D). In summary, 39 new candidate miRNAs that were differentially expressed between control and salt-stressed broccoli were identified by their read counts and confirmed by the stem-loop real-time RT-PCR.

### Target prediction and analysis of differential expression of miRNAs

To understand the biological mechanisms by which broccoli responds to salt stress, the putative target sites of miRNAs were identified by aligning miRNA sequences with the Expressed Sequence Tags (ESTs) of *B. rapa*, following the rules of target prediction suggested by Allen et al. [24]. A total of 836 and 527 mRNAs were predicted as targets of putative conserved miRNAs and putative novel miRNAs, respectively. In plants, the identification of mRNA targets is straight forward because most miRNAs and their target mRNAs have exact or nearly exact complementarity. Furthermore, the miRNA target sites of plants have been shown to be located primarily in the coding regions [25].

The putative target genes appeared to be involved in a wide variety of biological processes and molecular-genetic functions; therefore, the Gene Ontology (GO) database and the Kyoto Encyclopedia of Genes and Genomes (KEGG) Orthology (KO) database were used to analyze the datasets. Studies have shown that salt stress interferes with cell-cycle regulation at the transcriptional level, resulting in an adaptive growth response. Hormone signal transduction and sulfur metabolism are also vital for the improvement of stress tolerance in crop plants [26,27]. Interestingly, the results we obtained using the KO database indicated that genes involved in a diversity of cellular processes, such as the cell cycle, plant hormone signal transduction, and sulfur metabolism (Additional file 6: Table S4) were up-regulated in response to salt stress. Similarly, pathway annotation for the putative novel miRNAs that were up-regulated in response to salt stress indicated involvements in calcium reabsorption and in the cell cycle (Additional file 7: Table S5).

**Figure 4:**
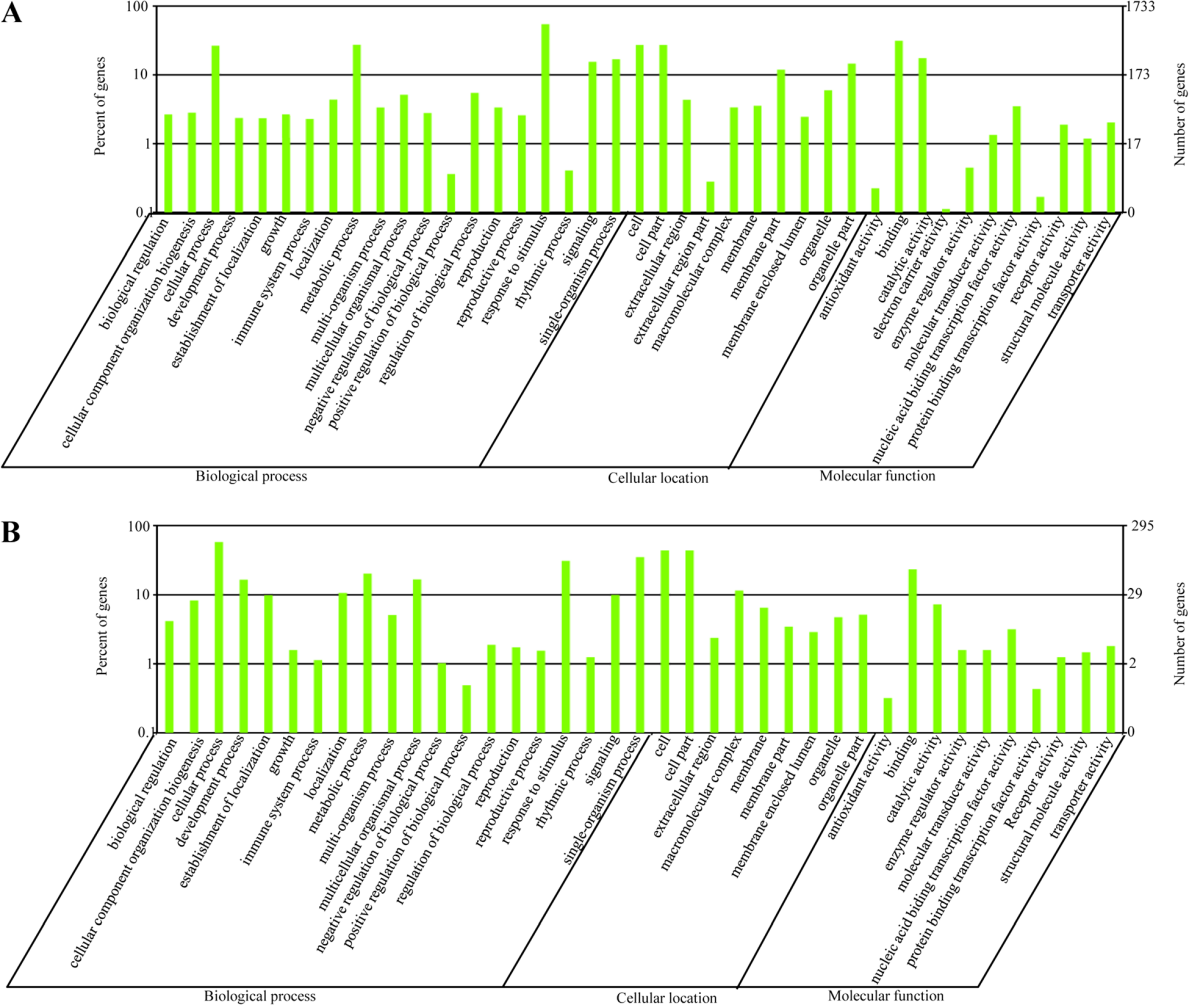
**Functional classification of miRNA targets according to the Gene Ontology (GO) program.** In this ontology, ‘cellular location’, ‘biological process’ and ‘molecular function’ are treated as independent attributes. GO classifications for putative conserved and putative novel miRNA targets are shown in the **upper panel** and **lower panel**, respectively.

The gene targets of both the putative conserved and the putative novel miRNAs were classified into three GO categories: cellular location, biological process and molecular function. For the cellular location category, large numbers of targets for putative conserved and putative candidate miRNAs were categorized under ‘cell’ and ‘cell part’, whereas ‘extracellular region’ comprised the smallest proportion (Figure 4A and B). For the biological process category, the majority were classified under ‘cellular process’, ‘metabolic process’ or ‘response to stimulus’. Under the molecular function category, the two most abundant sub-categories for both putative conserved and putative novel miRNAs were ‘binding’ and ‘catalytic activity’.

## Discussion

Salinity is an increasingly important agricultural problem. The metabolism and physiological integrity of plants are affected by salt stress and in recent years many studies have been devoted to understanding the molecular mechanisms of plant salt tolerance. Studies have confirmed that resistance to salt stress is associated with the use of compatible solutes by plants [28], and with ion transporters [29]. miRNAs are a class of non-coding sRNAs that are implicated in many developmental processes and in responses to various abiotic stresses, and which play pivotal roles in plant adaptation [1]. To date, in excess of 24,000 hairpin sequences and 30,000 mature sequences have been identified from plants, animals, unicellular organisms and viruses (miRBase); however, scant attention has been paid to the identification of miRNAs in broccoli. In the present study, we identified several million sRNA sequences in salt-stressed plantlets of broccoli, using high-throughput sequencing methods to identify miRNAs associated with physiology and metabolism under salt stress. A total of 42 putative known and 39 putative candidate miRNAs that were differentially expressed between control and salt-stressed broccoli were discovered according to their sequencing-reads numbers and confirmed by real-time RT-PCR. Finally, analysis of the predicted targets of the miRNAs using the GO and KO databases indicated that a range of metabolic pathways and biological processes known to be associated with salt stress were up-regulated in broccoli treated with salt.

The study of miRNAs using traditional methods is complex, and high-throughput sequencing methods now provide a rapid and efficient additional approach by which to identify and profile populations of sRNAs at different stages of plant development. Large numbers of non-conserved or species-specific miRNAs often accumulate in plants at lower levels than conserved miRNAs, and are therefore not easily revealed using traditional sequencing methods [3,30]. In the present study, the results indicated a range of sRNAs, of length 16–29 nt, in broccoli, with most of the unique sequence reads being 24 nt in length. Several plant species, including *A. thaliana*, *Citrus sativus* and *C. sinensis*, had been shown to contain substantially more 24-nt sRNAs than 21-nt sRNAs [31,32]; on the other hand, sRNAs populations with more 21-nt members than 24-nt were reported in *B. juncea* and in *Japanese apricot* with imperfect flower buds [32,33]. *B. juncea*, *B. napus* and broccoli all belong to the same genus; however, when broccoli sRNAs are compared to those of *B. juncea*, a striking difference exists, whereas little difference is observed when broccoli sRNAs are compared to those of *B. napus* [34]. Thus, we can conclude that the sRNA transcriptome is complex, and can be highly variable even between closely related plant species.

Generally speaking, length distribution analyses of sRNAs provide a helpful way to assess the composition of sRNA samples. Previous studies have shown that DCL1 mainly produces sRNAs that are 18–21 nt long. In contrast, the products of DCL2, DCL3 and DCL4 are 22 nt, 24 nt and 21 nt long, respectively. Furthermore, miRNAs are normally 21 nt or 22 nt long, whereas small interfering RNA (siRNA) are 24 nt long [35]. By this reasoning, therefore, our results imply that the most abundant sRNAs are miRNAs in broccoli and siRNAs that have been cleaved by DCL1 and DCL4.

Sequence information for broccoli miRNAs is entirely absent from miRBase, and furthermore there are fewer than 50 *B. rapa* miRNAs in this database. Therefore, the sRNA sequences were aligned instead against miRNA precursors/mature miRNAs of the Viridiplantae in miRBase. A search for conserved miRNAs revealed that the majority of these molecules, known already from *A. thaliana* and other species, were detectable in broccoli and were expressed at relatively high abundance. The patterns of expression of conserved miRNAs are variable in different plants. Previous studies had shown that miR159, miR166a, miR164, miR171f and miR168 were all detectable in *B.rapa*, and with relatively high numbers of reads, whereas members of the miR169 family showed low read numbers [30,34]. The findings of the present study were quite different; miR166a, miR168a and miR157d showed the highest values for copy number in control broccoli.

A previous report suggested that miR403, which was initially identified in *A. thaliana* and later found in *Populus trichocarpa*, was a dicot-specific miRNA and that miR437 and miR444 might be monocot-specific miRNAs [36]. On the other hand, Sunkar et al. indicated that miR390 was present in both monocots and dicots [25]. Our results provided a large-scale database against which to examine these suggestions. We detected both miR403 and miR390 in broccoli, whereas miR437 and miR444 were not detectable, thereby confirming the previous reports. Furthermore, the miR158 and miR391 sequences have been considered to be *A. thaliana*-specific [36]; however, we detected miR158a, miR158b, and miR391 in broccoli, each at an abundance of about 30,000 reads.

Although many plant miRNAs have been found to be conserved – for example, miR319, miR156/157, miR169, miR165/166 and miR394 have been found in more than 40 plant species [36] – studies have indicated that most miRNAs can be induced by environmental stresses. For example, miR399 is induced under low-phosphate conditions [37], miR395 increases upon sulfate starvation [8] and miR393 is up-regulated in response to cold, dehydration, NaCl and abscisic acid (ABA) stress [38]. A recent study showed that miR417 was transiently up-regulated in response to osmotic stress [39]. On the other hand, miR398 is down-regulated in response to oxidative stress. Several differentially regulated miRNAs have been identified in salt-stressed plants. For example, miR530a, miR1445, miR1446a-e and miR1447 were down-regulated during salt stress in *P. trichocarpa*, as measured by microarray analysis of known miRNAs, whereas miR482.2 and miR1450 were up-regulated [40]. In *A. thaliana*, miR156, miR158, miR159, miR165, miR167, miR168, miR169, miR171, miR319, miR393, miR394, miR396 and miR397 were all up-regulated in response to salt stress, whereas miR398 was down-regulated. Furthermore, miR169g and miR169n were also reported to be induced by high salinity [41]. Recently, a study of maize roots using miRNA microarray hybridization indicated that members of the miR156, miR164, miR167, and miR396 families were down-regulated by salt shock, whereas miR162 and miR168 were up-regulated [42]. Our result in broccoli indicated that, amongst the conserved miRNAs, miR393 and miR855 were the most strongly down-regulated in response to salt stress, whereas conserved miR838-5p and miR396a were the most strongly up-regulated. Of the putative novel miRNAs, miR37 showed the greatest degree of up-regulation in response to salt stress, whereas miR3 and miR34 showed the greatest degree of down-regulation. The differences between our findings and the reports relating to salt stress in *P. trichocarpa*, *A. thaliana* and maize roots [41] might be explained in part, of course, by differences in behavior between species but they might also reflect the use of different experiment methods: microarray analysis was used in some of the previous studies, whereas in the present study we used sequencing, which is more effective than microarray analysis for the detection of miRNAs, including novel miRNAs, that are expressed at low levels.

In the case of the human genome, more than a third of the genes have been predicted to be miRNA targets, and these targets would appear to be involved in a wide range of biological functions. In *A. thaliana*, miRNAs may target transcripts that encode proteins and transcription factors [43]. Our results indicated that a total of 836 and 527 mRNAs were targets of putative conserved miRNAs and putative novel miRNAs, respectively. These targets regulate a range of cellular processes, within the broad categories of cell-cycle regulation, plant hormonal signal transduction, and metabolism. However, there are several miRNAs without target genes; these could be the result of erroneous target predictions, or they might be low-abundance miRNAs with limited or no activity. It is also possible that miRNAs might exist that have no targets. Nevertheless, the KO and GO analyses revealed that many of the genes targeted by miRNAs in broccoli are related to salt stress, supporting the hypothesis that miRNAs play an important role in the response of broccoli to salinity.

## Conclusion

A comprehensive study of broccoli miRNAs in relation to salt stress has been undertaken. Our results provide new and significant data on the miRNA profile of broccoli. The differential regulation of miRNAs between control and salt-stressed broccoli indicates that miRNAs have a marked involvement in regulatory networks associated with salt stress. Further studies of gene function and regulation are now required in order to elucidate the precise mechanisms.

## Methods

### Plant material and growth conditions

Seeds of broccoli (*Brassica oleracea var. italic*) were obtained from Sakata Seed Corporation (Yokohama, Kanagawa, Japan). The seeds were cultivated and harvested as previously described [14]. Broccoli is moderately tolerant to salinity (40–60 mM NaCl), and therefore 80 mM NaCl was selected in order to study the effects of salt stress. The saline treatment was applied for 15 d, following 1 week of growth. At the end of this period, flowers from control broccoli (0 mM NaCl) and salt-stressed broccoli were immediately frozen in liquid nitrogen and stored at −80°C before use. In each case, samples were harvested and pooled from four individual flowers.

### sRNA library preparation and sequencing

Total RNA extractions from control broccoli and salt-stressed broccoli both of which were pooled from four individual flowers were performed using TRIzol™ reagent (Invitrogen, Carlsbad, CA, USA), following the manufacturer’s instructions. Total RNA quantity and purity were assayed with the NanoDrop ND-2000 spectrophotometer (Thermo Scientific, MA, USA) at 260/280 nm (ratio between 1.8 and 2.0). Moreover, the quality of total RNA was also analyzed by electrophoresis on a denaturing agarose gel and by Agilent Bioanalyzer (Agilent Technologies inc., Santa Clara, CA, USA). The total RNA extractions with RNA integrity number (RIN) value more than 8.0 and without DNA contamination were used for further study. The construction sRNA libraries and deep-sequencing were each performed by the Beijing Genomics Institute (BGI, Shenzhen, China). Briefly, the sRNA fraction, of length 18 to 30 nt, was extracted from a 15% denaturing polyacrylamide gel. The sRNAs were then ligated to a pair of Solexa adapters at their 5’- and 3’- ends, using T4 RNA ligase (New England Biolabs, Ipswich, MA, USA). Next, these sRNAs of length 70 to 90 nt were extracted from denaturing polyacrylamide gel and converted to DNA by RT-PCR. Finally, the purified PCR products were directly sequenced with a HiSeq 2000 Sequencing System (Illumina, San Diego, CA, USA), used according to the manufacturer’s protocol.

### sRNA analysis

The very basic figure from sequencing was converted into sequence data by the base calling step. Such sequence data called raw data or raw reads which was represented by four lines. The second line was the sequence. The fourth line represented the sequencing quality of this read. Each character in this line showed the sequencing quality of the base on the same position in the second line. The actual quality was the corresponding American Standard Code for Information Interchange (ASCII) value of the letter minus 64. The quality of HiSeq sequencing ranged from 0 to 41. This quality would be used in the criteria for filtering out low quality reads. The sequence tags from the HiSeq sequencing were processed via a data-cleaning pipeline developed by Beijing Genome Institute (BGI, Beijing, China) in order to remove the tags with: any N bases, more than 4 bases whose quality score was lower than 10 and more than 6 bases whose quality score was lower than 13, and those that were too small (with length shorter than 18 nt), as well as to remove the adapter sequences from the tags [9]. The remaining sequences generated from Illumina HiSeq and went through data cleaning process were called clean sRNA reads, and they were mapped to the *B. rapa* genome using SOAP to analyze their expression and their distribution within the genome. The program was performed using the following parameters: soap -v 0 -r 2 -m 0 -a clean.fa -d ref_genome.fa.inedx -o match_genome.soap. Sequences with perfect match or one mismatch were retained for further analysis. We then used the basic local alignment search tool (BLAST) (http://blast.ncbi.nlm.nih.gov/Blast.cgi) search of sRNAs against the Rfam database in order to remove non-coding RNAs, such as rRNA, tRNA, snRNA and snoRNA. The remaining sequences were then used for further characterization.

### Identification of conserved and novel miRNAs

Following initial processing, homologs of known miRNAs and the remaining non-annotated sRNAs were used to identify miRNAs. Since broccoli miRNA sequences were not available in miRbase, we instead, aligned the sRNA tags against miRNA precursor/mature miRNA sequences of the Viridiplantae belonging to 52 plant species in miRBase (miRBase release 20.0, June, 2013, http://mirbase.org/), in order to identify sequences and miRNA families (not individual species) that were represented in the samples. First, clean data were aligned against the miRNA precursor/mature miRNA sequences, allowing two mismatches or gaps to take account of inter-species differences; secondly, for each mature miRNA family, the miRNAs with the highest expression levels were selected as a cluster to create a temporary miRNA database; thirdly, total miRNA expression levels were calculated by summating the clean reads that could align to the temporary miRNA database within two mismatches; finally, sequences identified as miRNAs but which could not fold hairpin structure were categorized as pseudo-miRNAs [44]. Moreover, an adaptive thresholding method using Kolmogorov-Smirnov (KS) statistics was employed to achieve a threshold value, according to the report of Winston et al. [45]. Interestingly, our results indicated that transcripts with a read count of more than 38 are thus deemed to be significantly different from noise both in control and salt-stressed broccoli.

The prediction of novel miRNAs was carried out using a new algorithm termed ‘MIREAP’, which could identify novel miRNA candidates from their possession of a canonical hairpin structure, and which was adequately supported by sequencing data (http://sourceforge.net/projects/mireap), developed by the BGI. The following parameters were applied. It includes minimal miRNA length (18), maximal miRNA length (25), minimal miRNA reference sequence length (20), maximal miRNA reference sequence length (23), maximal copy number of miRNAs on reference (20), maximal free energy allowed for a miRNA precursor (−18 kcal/mol), maximal space (300), minimal mature pair (16), maximal mature bulge (4), maximal duplex asymmetry (4), and flank sequence length (20). Structures that conformed to previously established criteria were considered as candidate novel miRNAs [46]. The stabilities of the candidate pre-miRNAs were assayed using Mfold (http://sourceforge.net/projects/mireap). Since these miRNAs were mapped to genomic loci on *B. rapa* chromosomes, we denoted then as ‘putative novel miRNAs’of broccoli.

### Analysis of differential read counts of miRNAs between salt-stressed broccoli and control broccoli

To compare the differential read counts of miRNAs between salt-stressed broccoli and control broccoli, frequencies of miRNA read counts were normalized as transcripts per million and the normalization of miRNA expression levels between the two broccoli libraries was carried out based on the following formula: normalization of miRNA read counts (NC) = (Actual miRNA count / Total count of clean reads) × 10^6^. If the normalized association value of a given miRNA is zero, its association value was modified to 0.01; if the miRNA gene expression of two samples was < 1, on account of low expression, the miRNA was neglected in the analysis of differential expression. Then, the fold-change and FDR were calculated from the normalized association using the following equations: Fold-change = log2 (NC of salt-stressed broccoli / NC of control broccoli); FDR was calculated using the formula below [32], where: N_1_ and N_2_ represent the total counts of clean reads of a given miRNA in the sRNA libraries of control and salt-stressed broccoli samples, respectively; and *x* and *y* represent the normalized expression levels of a given miRNA in the sRNA libraries of control and salt-stressed broccoli samples, respectively.

$$ FDR\left(x/y\right)={\left({N}_2/{N}_1\right)}^y{\frac{\left(x+y\right)!}{x!y!\ {\left(1+{N}_2/{N}_1\right)}^{\left(x+y+1\right)}}}_{D\ \left(y\ \ge\ {y}_{\max }x\right)={\displaystyle \sum_{y\ge {y}_{\max}}^{\infty } FDR\left(y\backslash x\right)}}^{C\left(y\ \le\ {y}_{\min }x\right)={\displaystyle \sum_{y = 0}^{y\ \le {y}_{\min }} FDR\left(y\backslash x\right)}} $$

### miRNA quantification by real-time RT-PCR

To validate the results from the bioinformatics-based analysis, stem-loop real-time RT-PCR was performed. Total RNA was first extracted from other three individual control flowers and salt-stressed flowers of broccoli, which were not the same with RNA used for sRNA analysis, using TRIzol™ reagent. Then, 1 μl of the stem-loop RT primer (1 μM) and 2 mg of total RNA were used in first-strand cDNA synthesis with an iScript™ cDNA synthesis kit (Bio-Rad, Hercules, CA, USA). Real-time RT-PCR was carried out using 0.5 ml of cDNA and specifically designed forward primers for each individual miRNA, together with the universal reverse primer, on a Mx3005P qPCR System (Agilent Technologies, Santa Clara, CA, USA), using an iQ™ SYBR® Green Supermix kit (Bio-Rad). The protocol for stem-loop real-time PCR was as follows: initial denaturation at 95°C for 10 s, followed by 40 cycles of 10 s at 95°C and 20 s at 55°C. At the end of each PCR reaction, a melting curve was determined, and only samples that displayed a one-peak melting curve at the correct annealing temperature were used for subsequent analysis. The threshold was automatically set and the threshold cycle (Ct value) was recorded. The real-time RT-PCR reactions were repeated at least three times to ensure statistical rigor. Finally, the miRNA expression was calculated from three independent biological replicates. SnRNA U6 was used as an internal control. Fold changes were calculated by relative quantification. The 2^△△^*C*_T_ method has been used to calculate relative changes in gene expression determined from real-time quantitative PCR experiment [47] using the following equations: ^△△^*C*_T_ = (C_T, miRNA_ − C_T, U6_)_Salt-stressed broccoli_ − (C_T, miRNA_ − C_T, U6_) _Control broccoli_, where, the *C*_T_ values were directly provided from real-time PCR instrumentation. Statistical differences between groups were assessed using the independent two-sample t-test. Statistical analysis was performed with SPSS statistical package (V15.0). Moreover, the products of PCR were also confirmed by electrophoresis sequencing and denaturing gel electrophoresis.

### Prediction of miRNA targets

To explore the biological functions of miRNAs in broccoli, putative target sites were identified by aligning the miRNA sequences to the ESTs of *B. rapa*, and adopting the alignment criteria suggested by Allen at al. [46] and Schwab et al. [48]. Briefly, the criteria were defined as follows: i) ≤ 4 mismatches between the miRNA and the target (a G-U base mismatch counts as 0.5 mismatches); ii) no adjacent mismatches in positions 2–12 of the miRNA/target duplex (counting from the 5’ of the miRNA); iii) ≤ 2 adjacent mismatches in the miRNA/target duplex; iv) no mismatches in positions 10–11 and no more than 2.5 mismatches in positions 1–12; and v) the Minimum Free Energy (MFE) of the miRNA/target duplex should be ≥ 75% of the MFE of the miRNA bound to its perfect complement.

### KEGG pathway and GO-enrichment analyses

In order to investigate further the biological functions of miRNAs and their targets, KEGG pathway analyses were undertaken on the predicted miRNA-target genes. These putative miRNA-target sequences were used as queries against the KEGG database. AGO-enrichment analysis was also undertaken for the target gene candidates. Categories relating to biological processes, cellular components and molecular functions were displayed for the miRNA-target genes. The results revealed those cellular functions that were related significantly to the predicted gene targets of the miRNAs.

### Statistical analysis

Statistical analysis was performed with the SPSS statistical package (v15.0). The results of real-time RT-PCR were repeated three times and data from three independent biological replicates are presented as the mean ± standard deviation (SD). Statistical differences amongst groups were assessed using the independent two-sample t-test. *P*-values < 0.05 were considered statistically significant.

## Additional files

Additional file 1: Table S1. Summary of sRNA sequencing data. Additional file 2: Table S2. Mapping statistics: sRNAs to genome. Additional file 3: Figure S1. Total and unique clean reads annotated into categories Control broccoli and salt-stressed broccoli are shown in (A) and (B), respectively. Total reads and unique reads are presented in the left panel and right panel, respectively. Additional file 4: Table S3. The differential expression of putative conserved miRNAs between the two libraries. Additional file 5: Figure S2. Prediction of the secondary structures of all new candidate miRNAs in salt-stressed broccoli A total of 43 putative novel miRNAs are shown; see also Figure 3B. Additional file 6: Table S4. Pathway annotations for most of targets of putative conserved miRNAs in salt-stressed broccoli. Additional file 7: Table S5. Pathway annotations for most of targets of putative novel miRNAs in salt-stressed broccoli.
