# Supplementary figures and images for: Identification and characterization of microRNAs related to salt stress in broccoli, using high-throughput sequencing and bioinformatics analysis
Source: BMC Plant Biol. 2014 Sep 3;14:226. doi: 10.1186/s12870-014-0226-2 (PMC4167151; doi:10.1186/s12870-014-0226-2)

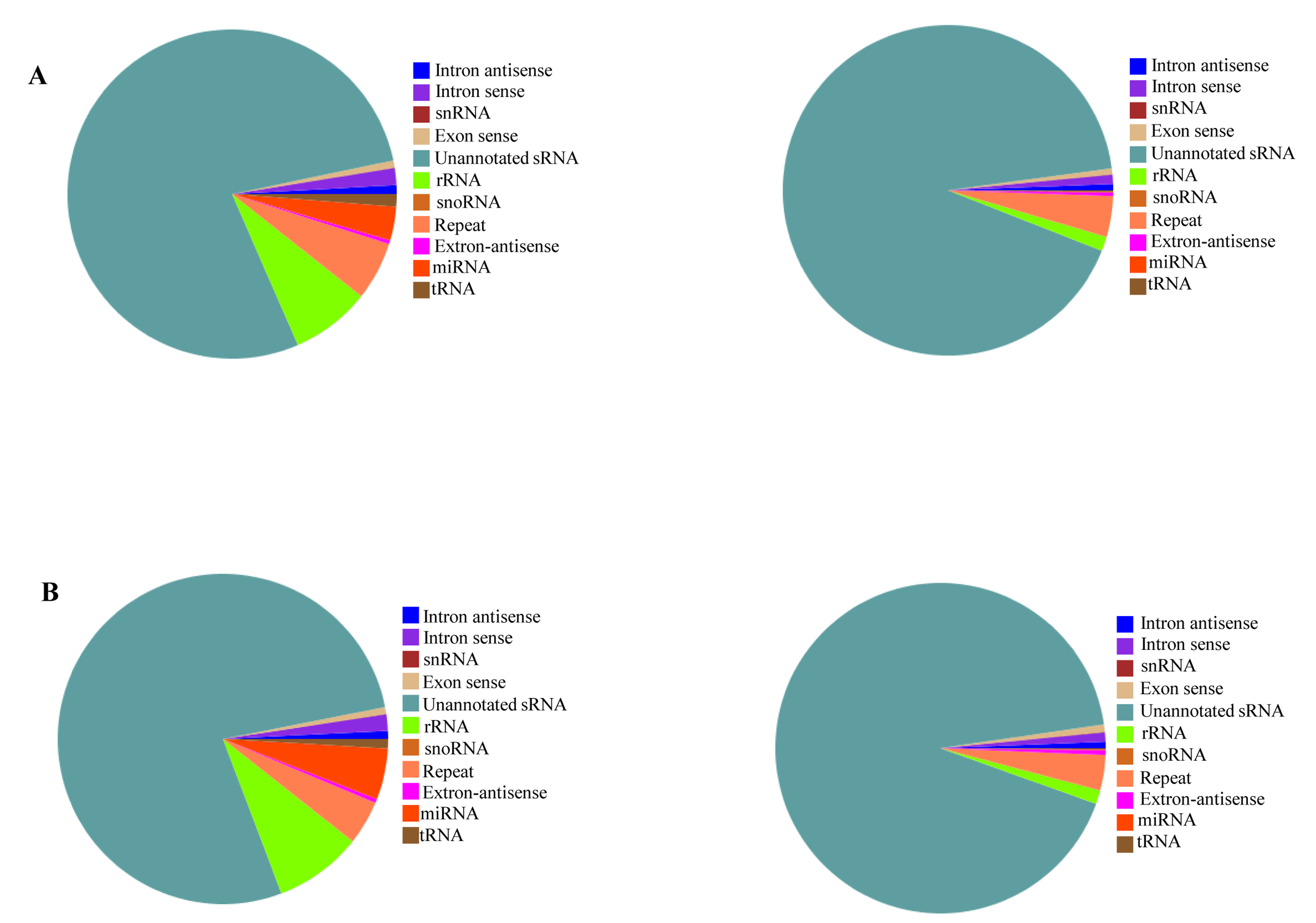

Supplement: Additional file 3: Figure S1. — Total and unique clean reads annotated into categories Control broccoli and salt-stressed broccoli are shown in (A) and (B), respectively. Total reads and unique reads are presented in the left panel and right panel, respectively. [file 12870_2014_226_MOESM3_ESM.tiff]

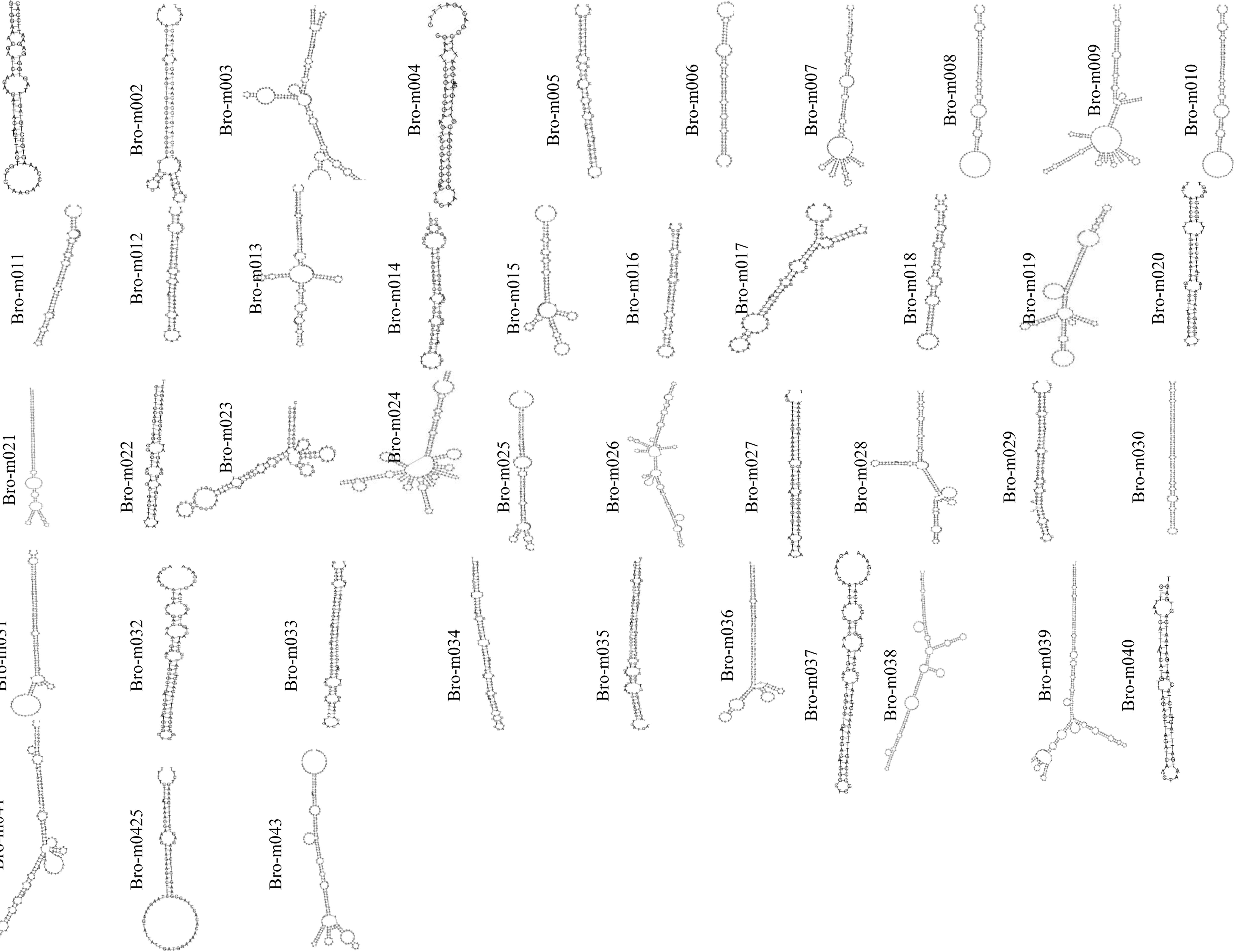

Supplement: Additional file 5: Figure S2. — Prediction of the secondary structures of all new candidate miRNAs in salt-stressed broccoli A total of 43 putative novel miRNAs are shown; see also Figure 3B. [file 12870_2014_226_MOESM5_ESM.tiff]
